# Effects of *Panax* ginseng-containing herbal plasters on compressed intervertebral discs in an *in vivo* rat tail model

**DOI:** 10.1186/1749-8546-8-4

**Published:** 2013-02-18

**Authors:** Daniel H K Chow, Alon Lai, Fuk-Hay Tang, Mason C P Leung

**Affiliations:** 1 Interdisciplinary Division of Biomedical Engineering, The Hong Kong Polytechnic University, Hung Hom, Kowloon, Hong Kong, SAR, PR China; 2 Department of Rehabilitation Sciences, The Hong Kong Polytechnic University, Hung Hom, Kowloon, Hong Kong, SAR, PR China

## Abstract

**Background:**

*Tienchi* (*Panax notoginseng*) has been used in conservative treatments for back pain as a major ingredient of many herbal medicines. This study aims to investigate the effects of a herbal medicine containing *tienchi* on compressed intervertebral discs in rats.

**Methods:**

Using an *in vivo* rat tail model, intervertebral disc compression was simulated in the caudal 8–9 discs of 25 rats by continuous static compression (11 N) for 2 weeks. An herbal medicine plaster (in which the major ingredient was *tienchi*) was externally applied to the compressed disc (n=9) for three weeks, and held in place by an adhesive bandage, in animals in the Chinese Medicine (CM) group. The effect of the bandage was evaluated in a separate placebo group (n=9), while no intervention with unrestricted motion was provided to rats in an additional control group (n=7). Disc structural properties were quantified by *in vivo* disc height measurement and *in vitro* morphological analysis.

**Results:**

Disc height decreased after the application of compression (*P* < 0.001). The disc height decreased continuously in the control (*P* = 0.006) and placebo (*P* = 0.003) groups, but was maintained in the CM group (*P* = 0.494). No obvious differences in disc morphology were observed among the three groups (*P* = 0.896).

**Conclusion:**

The *tienchi*-containing herbal plaster had no significant effect on the morphology of compressed discs, but maintained disc height in rats.

## Background

Back pain affects >50% of the general population [1,2]. Sources of back pain are highly associated with innervated spinal structures (*e.g.*, discs, muscles, ligaments and facets), and about 40% of cases of back pain are attributed to the intervertebral discs [3]. Intervertebral disc degeneration has also been shown to be significantly associated with back pain [4]. Moreover, the effects of therapeutic interventions are transient, with potential recurrent back pain, if a degenerated disc is not restored [4]. Thus, regeneration of degenerated discs is an important therapeutic strategy for back pain.

*Tienchi* (*Panax notoginseng*) is a common herb used for preparing herbal plasters for pain relief, especially relief of back pain, in Chinese Medicine (CM) [5-7]. *Panax notoginseng* can facilitate blood circulation and reduce inflammation [6] by inhibiting the production of tumor necrosis factor-α (TNF-α), interleukin-1β (IL-1β), inducible nitric oxide (iNO) and matrix metalloproteinase-13 (MMP-13) in different experimental models [7-11]. This regulation of cytokines, iNO and matrix-degrading enzymes could control the process of disc degeneration, or contribute to restoration of the degenerated disc [12-16].

According to the manufacturer, the herbal remedy is made from different herbs and a high performance liquid chromatography analysis of the herbal remedy identified ginsenoside Rb1 extracted from tienchi as one of the major effective constituents [17].

This study aims to investigate the effects of a *tienchi*–containing herbal plaster on compressed intervertebral discs in rats.

## Methods

### Experimental design

Skeletal mature male Sprague–Dawley rats (n = 25) (4–5 months old) [18] were arbitrarily allocated to one of three experimental groups: (A) CM, (B) placebo, and (C) control. Ethical approval (ASESC 07–8) was obtained for all animal procedures from the Animal Subjects Ethics Sub-Committee of the Hong Kong Polytechnic University.

**Figure 1:**
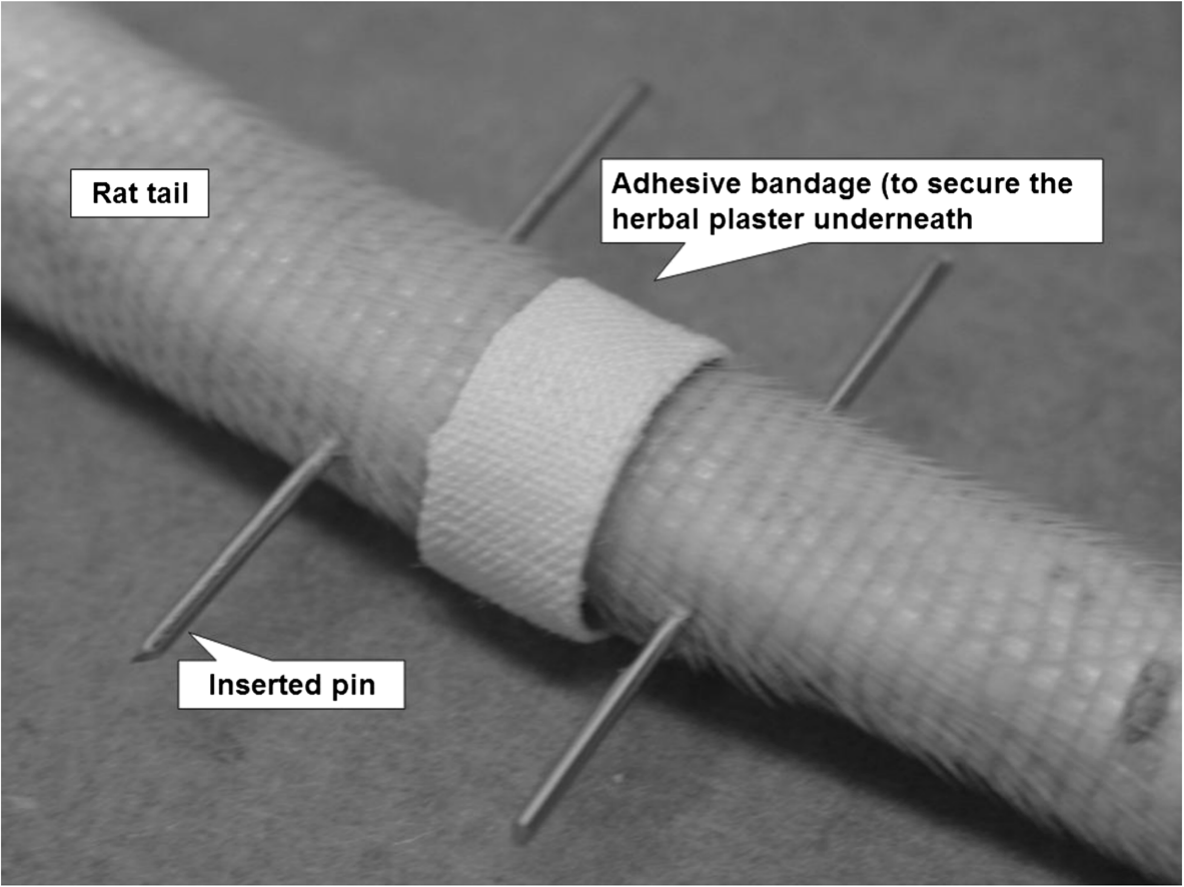
**Rat tail with two inserted pins at the caudal 8**^**th **^**and 9**^**th **^**vertebral bodies.** The herbal medicine plaster was applied to the degenerated disc externally, located at the mid-point between the inserted pins, and held in place by an adhesive tape.

Disc degeneration was simulated in rats’ caudal 8–9 intervertebral discs by continuous static compression of 11 N for 2 weeks *via* two stainless steel pins inserted into the adjacent vertebral bones [19-23]. Subsequently, the rats in the CM group were unloaded and a traditional herbal plaster (Tienchi Huo Luo Bruise Analgesia Plaster, Guangzhou Pharmaceutical Import & Export Corporation, P.R. China) was externally applied to the compressed disc for the following 3 weeks (*i.e.*, days 18–38), held in place by an adhesive bandage (Figure 1). The effect of the adhesive bandage was evaluated in the placebo group. The bandages and plasters were replaced every day for reasons of hygiene. No intervention with unrestricted motion was provided to the rats in the control group after the application of compression. The heights of caudal 8–9 discs were measured on days , 18 and 39 after pin insertion. Disc heights were also measured after one day of resting to eliminate any transient changes [21-23]. After disc height measurement on day 39, the rats were sacrificed and their discs were harvested for morphological analysis.

### Pin insertion

The animals were anesthetized with an intraperitoneal injection of ketamine (70 mg/kg, Alfasan International B.V, Woerden, The Netherlands) and xylazine (7 mg/kg, Alfasan International B.V, Woerden The Netherlands) during the operation. Two sterilized stainless pins (0.85 mm in diameter) (The Industrial Centre, The Hong Kong Polytechnic University, Hong Kong) were inserted into the mid-transverse plane of the 8th and 9th caudal vertebral bodies using a variable speed drill with the aid of a specially designed alignment jig (Figure 1) [19-22]. The locations for pin insertion were determined based on a pre-operative radiograph (Siemens Multix Pro General Diagnostic X-ray Unit, Siemens AG Medical Engineering, Germany), and a post-operative radiograph was taken to confirm the alignment and locations of the inserted pins [19-22]. The wound sites were disinfected daily with 75% ethanol and iodine solution to prevent pin tract infection. No intervention was applied over the first three postoperative days for wound healing. The general health of the animals was monitored throughout the experiment *via* their body weights.

### Static compression

Continuous static compression of 11 N was applied to the caudal 8–9 discs *via* the inserted pins for 2 weeks to simulate disc degeneration [21-23]. The compressive force was applied by a custom-made spring-loaded device attached to the inserted pins. The compressive loading was controlled by two springs, which were calibrated prior to the experiment. The strains of the springs were monitored every day, with adjustment to ensure a consistent loading [21-23].

### Herbal medicine plaster

The herbal plaster (1 mm in thickness) contained a mixture of Chinese medicinal herbs with the major ingredient being *tienchi*. The herbal medicine was prescribed by a registered CM practitioner. Prior to application, the plaster was cut into small pieces (5 × 35 mm) and circumferentially surrounded the rat tail. The position of the plaster was secured by an adhesive bandage (6 × 45 mm) (Figure 1).

### *In vivo* disc height measurement

The rat caudal 8–9 disc height was quantified *in vivo* by radiographic methods using an image intensity adjustment technique [21-24]. The tails of anesthetized rats were aligned straight on a specially designed acrylic V-block. A longitudinal radiograph was taken with a standardized protocol of 50 kVp and 5 mAs and the film was digitized for image analysis. The intensity of the image was adjusted with reference to an aluminum block attached to the V-block using image editing software (Adobe Photoshop 5.0, Adobe Systems Incorporated, San Jose, USA) to control for differences in intensities between images and to enhance the contrast of the vertebral boundary for measurement. The disc height was determined using image analyzing software (Scion Image Beta 4.02, Scion Corporation, Maryland, USA). A reference line was drawn through the middle of the longitudinal axis of the caudal 8th and 9th vertebral bodies and multiple lines parallel to the reference line were drawn towards the two lateral margins of the vertebral bones until no overlapping was observed. The intervertebral distances along the longitudinal lines in the center region (*i.e.*, half of the number of parallel lines) were measured three times and averaged to determine the disc height. All measurements were referenced to the length of the aluminum block. This disc height measurement technique was shown to be highly reliable in a rat tail model with a high intra-rater correlation coefficient (ICC) of 0.996 [24].

### *In vitro* morphological analysis

After disc height measurement on day 39, the rats were sacrificed by overdose with anesthetics. After removal of the surrounding skin, soft tissues and inserted pins, spinal cord segments from caudal 6th to 11th were harvested by cutting through the caudal 5–6 and 11–12 discs. The segments were divided into three sub-segments (namely, caudal 6–7, 8–9 and 10–11) by cutting through the caudal 7–8 and 9–10 discs. The caudal 8–9 disc was the target disc, while the caudal 6–7 and 10–11 discs were used as control discs for comparison. All sub-segments were fixed in 4% neutral buffered formaldehyde (Leica Microsystems GmbH, Wetzlar, Germany) for 2 days and decalcified in 14% ethylene-diamino-tetraacetic acid (Sigma, St. Louis, MO, USA) for 8 weeks. After processing, they were embedded in paraffin and then sectioned along the sagittal plane through the nucleus pulposus (NP) at a thickness of 4 μm. All sections were stained with hematoxylin and eosin (Leica Microsystems GmbH, Wetzlar, Germany) for cellular constituents and Safranin-O (Sigma, St. Louis, MO, USA) for proteoglycans [21,23,25]. Disc morphology was examined using an optical microscope (Nikon Research Upright Microscope Eclipse 80i, Tokyo, Japan) and quantified using a four-category histological grading scale proposed by Masuda *et al.*[25], including (a) the annulus fibrosus; (b) the border between the annulus fibrosus and the NP; (c) the cellularity of the NP; and (d) the matrix of the NP. Each category was graded from 1 to 3 with grade 1 for discs with normal morphology and grade 3 for characteristics of severe degeneration. Thus, the overall scores for normal discs and severely degenerated discs were 4 and 12, respectively.

### Statistical analysis

Data are presented as means ± standard deviation (SD). The changes in disc height (between Days 4–18 and Days 18–39) among different groups were compared by one-way analysis of variance with group as a between-subject factor. Bonferroni-corrected post-*hoc* tests were performed for further comparisons between individual groups. The histological grades of the target disc (*i.e.*, caudal 8–9) were compared to those of control discs (*i.e.*, caudal 6–7 and 10–11) using a non-parametric Friedman test with disc level as a within-subject factor, and non-parametric Wilcoxon tests were performed for post-*hoc* comparisons between individual disc levels. The grades of target disc among different groups were compared by Kruskal-Wallis test with group as a between-subject factor and Mann–Whitney tests were performed for post-*ho*c comparisons between individual groups. All statistical analyses were conducted using statistical software (SPSS v.18, SPSS Inc., Chicago, USA) with the threshold for significance set at 0.05.

## Results

### Animal condition

All rats completed their individual experimental protocols. Average body weights for CM group are: 446±26 g, 453±29 g, 447±33 g on days 4, 18 and 39 respectively. For placebo group, they are: 452±31 g, 464±40 g, 457±41 g respectively. For control group, they are: 399±39 g, 430±43 g, 439±41 g respectively. No pin tract infection or other complication was found at any pin insert region. The animals could also tolerate the spring-loaded compressive device well, with no observable restriction of their tail movement, except the compressed segment. Moreover, no infection or other complication was observed in any rat tail after the application of CM and/or the adhesive bandage.

### Disc height

**Figure 2:**
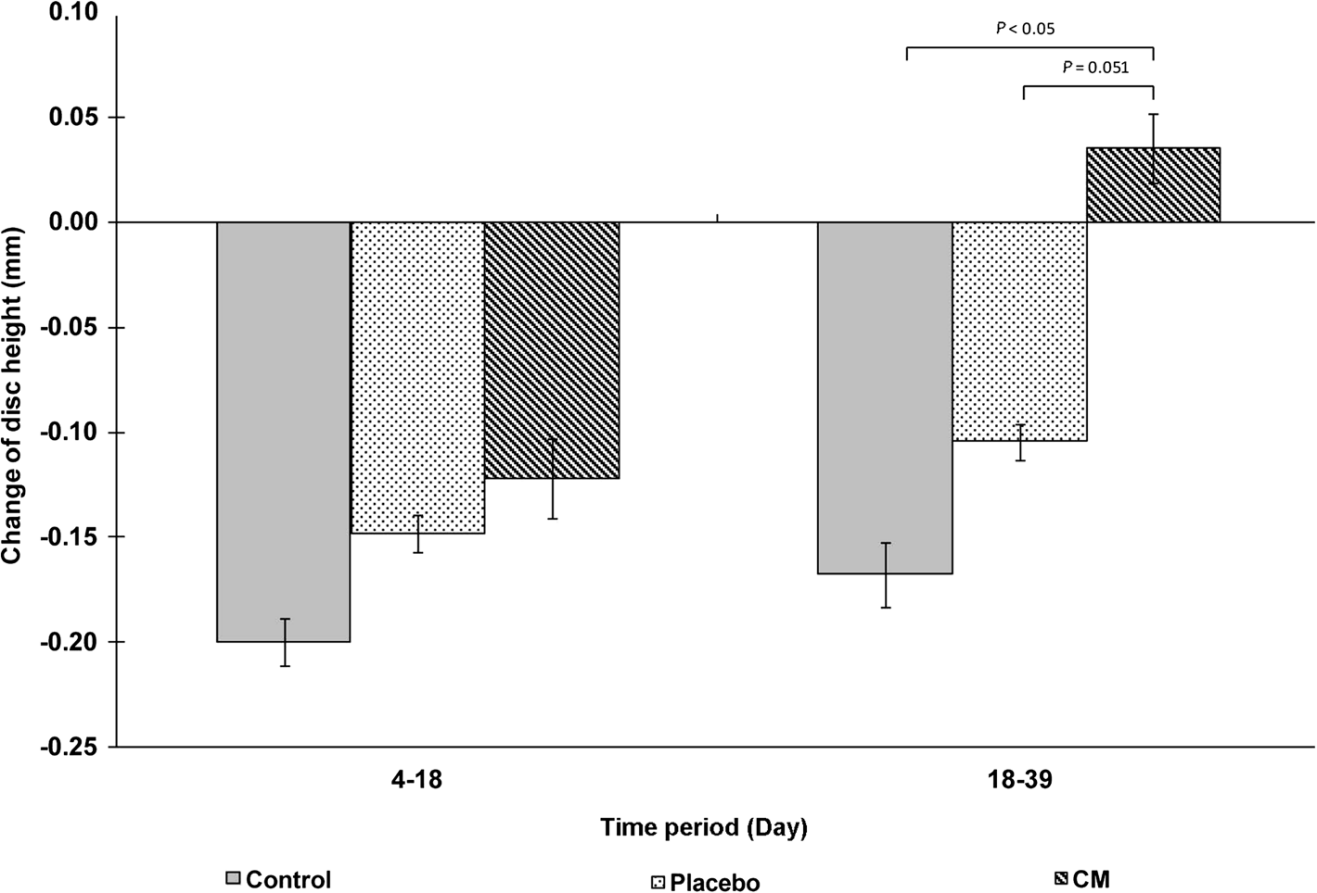
**Changes in disc height between day 18 and day 39 among the three experimental groups.** A negative value indicates a decrease in disc height, while a positive value indicates an increase in disc height. The standard deviations are shown as error bars, and significant differences are marked.

**Figure 3:**
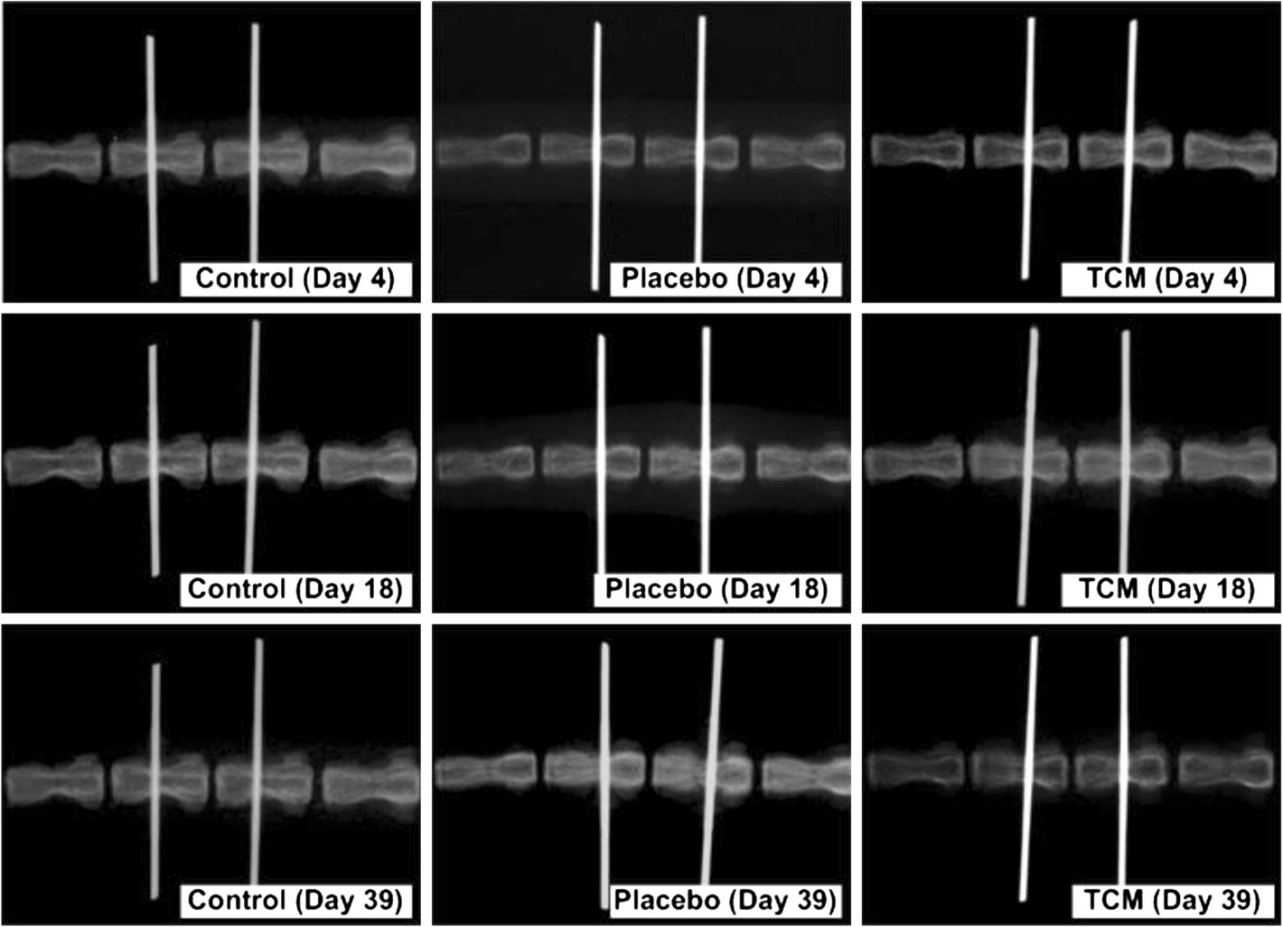
Radiographs of rat caudal 8–9 intervertebral discs of the three experimental groups showing the changes in disc height at different time points.

After the application of static compression, all disc heights decreased and the changes in disc height after compression were not significantly different among the three groups (*P =* 0.455). The disc height decreased continuously in the control (*P* = 0.006) and placebo (*P* = 0.003) groups, while disc height was maintained in the CM group (*P* = 0.494). As a result, the change in disc height in the CM group was significantly different from those in the control (*P* = 0.006) and placebo (*P* = 0.051) groups (Figures 2 and 3).

### Morphological analysis

**Figure 4:**
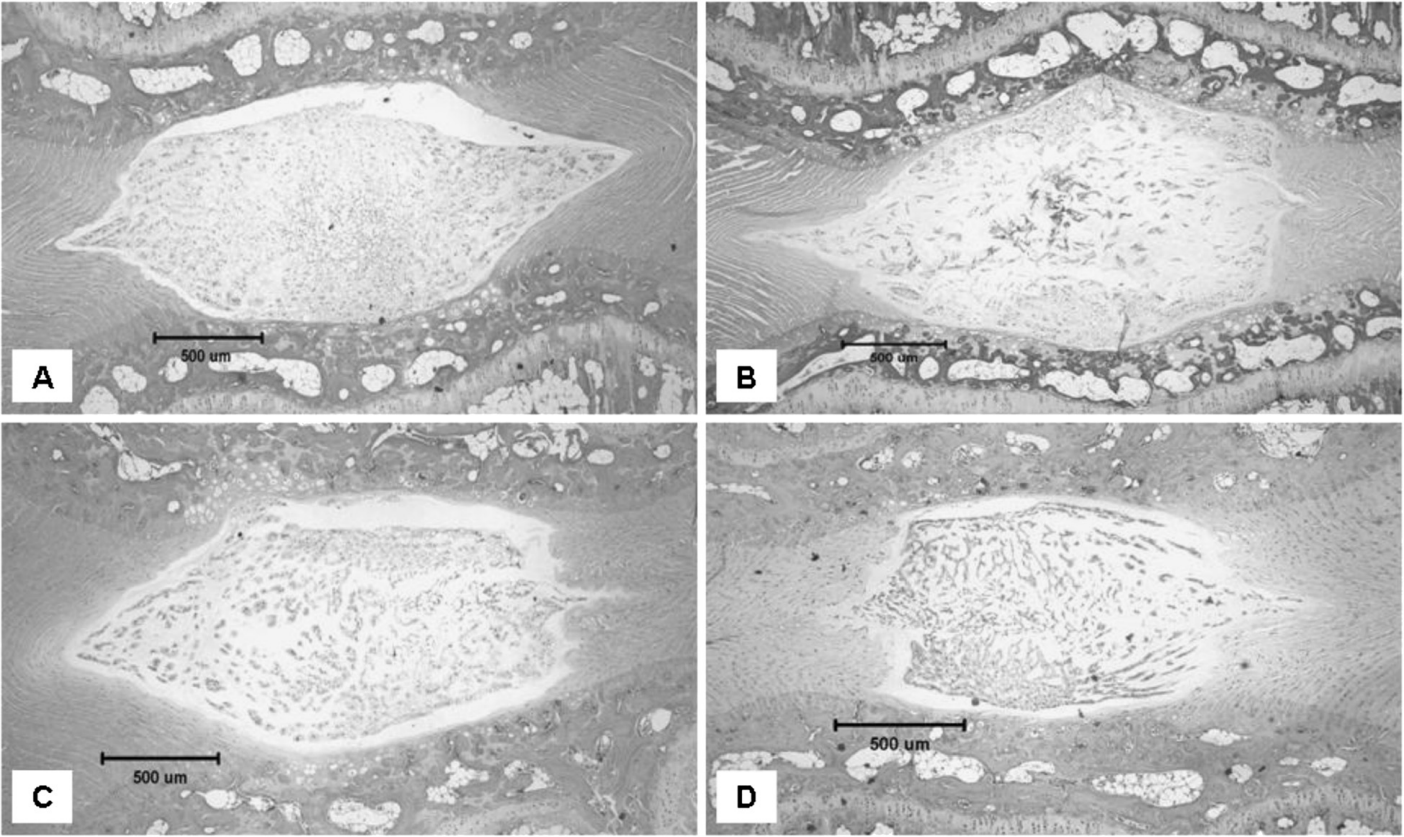
Tissue sections of rat caudal intervertebral discs from the different experimental groups stained by hematoxylin and eosin, including A) control normal disc and degenerated discs in B) the control group, C) the placebo group and D) the CM group.

**Figure 5:**
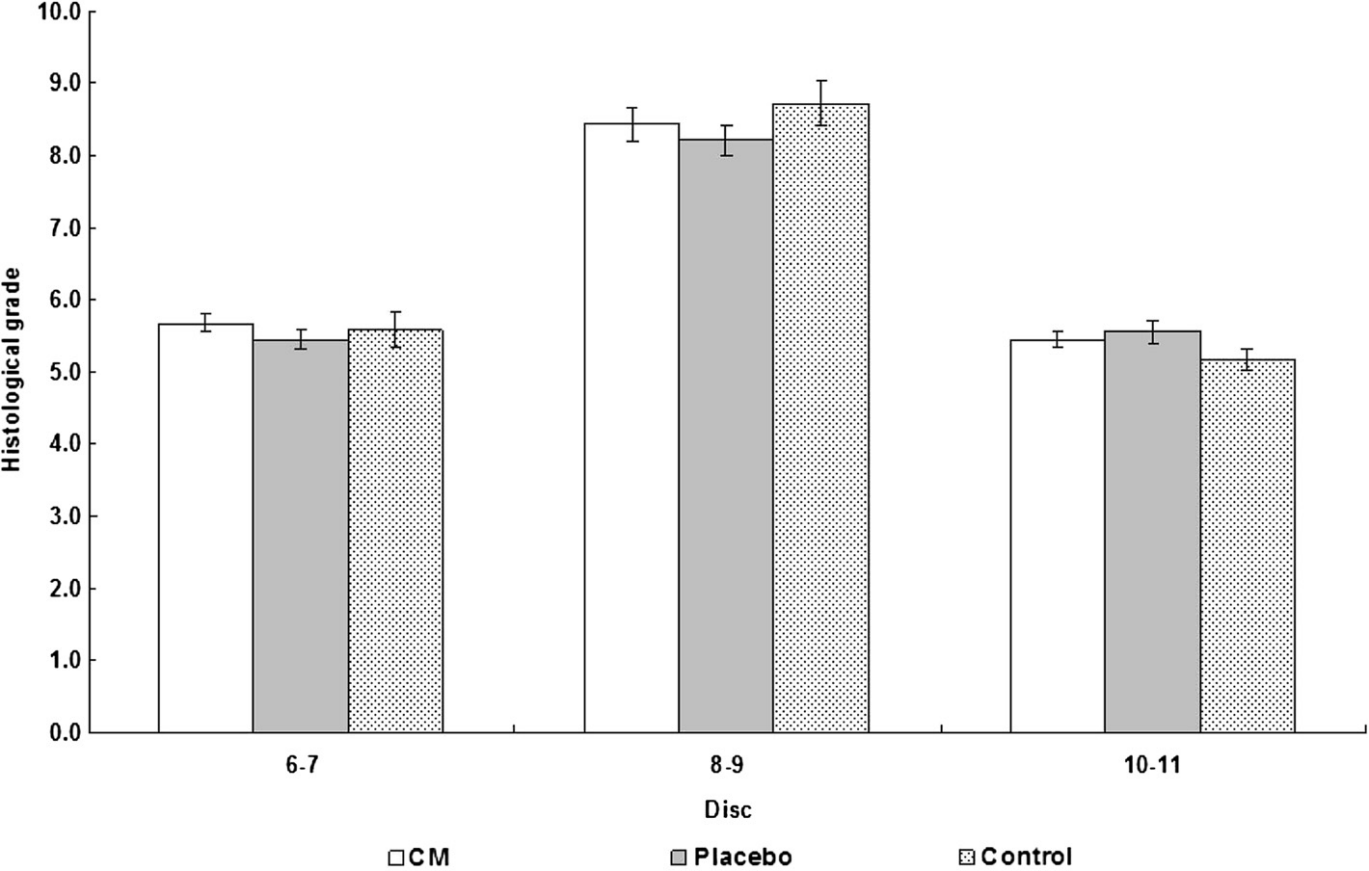
**Histological grades of the target disc (*i.e.*, caudal 8–9) and control discs (*i.e.*, caudal 6–7 and 10–11) of the three experimental groups.** The standard errors are shown as error bars and significant differences relative to the corresponding target disc are marked with asterisks.

A total of 75 rat caudal discs were obtained for morphological analysis. One of the 75 specimens was excluded from the study because of tissue damage that occurred during the tissue processing procedures. The control discs showed normal morphological characteristics with an oval-shaped NP, round nuclear cells, a U-shaped well-organized annulus fibrosus, and a clear nuclear boundary with no inward bulging annular lamellae (Figure 4). Signs of degeneration were observed in the control and placebo groups after 3 weeks. Histologically, the NP was irregular in shape with inward bulging annular lamellae. The nuclear cells became spindle-like in shape. The lamellar architecture of the inner and middle annulus became less organized. The boundary of the NP became less obvious (Figure 4). However, there was no sign of regeneration following 3 weeks of herbal medical treatment. Moreover, there were no significant differences in histological grade among the three groups (*P* = 0.896), although the scores for compressed discs were significantly higher than those for control discs (both caudal 6–7 and caudal 10–11 discs) (*P* < 0.001; Figure 5).

## Discussion

Because disc degeneration is associated with back pain [4], the effects of therapeutic interventions are anticipated to be transient if degenerated discs cannot be restored. This study aimed to study the therapeutic effects of *tienchi*-containing herbal plasters on compressed intervertebral discs in rats.

Degeneration was simulated in rat caudal discs by the application of continuous static compression for 2 weeks (*i.e.*, from day 4 to 17) with evidence of decreased disc height. During the following three weeks (*i.e.*, day 18 to 38), control group rats were allowed to perform unrestricted motion, while a herbal medicine plaster was applied to the tails of CM group rats at the level of the compressed disc, where it was held in place by an adhesive bandage. Because the circumferential circulation in tails and motor behavior of rats might be affected by the tension of the bandage, the effect of the bandage was studied in the placebo group. The disc height continuously decreased from day 18 to 38 in the control and placebo groups. The results of morphological analysis showed that the compressed discs in animals in both groups could not be restored, and that the effect of the bandage on the compressed disc was minimal. In the CM group, after 3 weeks of herbal plaster application, although no improvement in morphological characteristics was observed, the disc height was maintained, revealing that the herbal medicine plaster might potentially benefit the compressed disc. The lack of any significant change in disc morphology might be due to the short therapeutic period of three weeks. Kroeber *et al.*[26] demonstrated that morphological signs of disc regeneration were associated with the duration of disc distraction, with more pronounced changes occurring after 28 days of distraction compared with those occurring after 7 days of distraction. The maintenance of disc height in the CM group might have resulted from the vasodilative effect of *Panax notoginseng*[6,27]. The vasodilation of circumferential blood vessels increases vascular permeability and blood flow [28], which would probably increase the intradiscal water content and subsequently prevent further decreases in disc height. Furthermore, the lack of obvious morphological changes revealed that the change in disc height is multi-factorial, and that a favorable change in disc height does not necessarily indicate a positive change in disc morphology. Extracellular matrix components (particularly proteoglycans, collagen and matrix-degrading enzymes) are also important with respect to changes in disc height. Further biochemical analysis of the contributions of extracellular matrix components to disc height is required to explore the beneficial effects of herbal medicine plasters comprising *tienchi*.

The positive effect on disc height also might have resulted from the anti-inflammatory effects of *Panax notoginseng*. Chang *et al.*[7] found that *Panax notoginseng* significantly inhibited the production of TNF-α, IL-1β, iNO and MMP-13 in human monocytic cells, human chondrosarcoma cells and mouse macrophage cells. Rhule *et al.*[9] found that *Panax notoginseng* inhibited the production of TNF-α and IL-1β in cultured macrophages. In addition, Jin *et al.*[10] showed that *Panax notoginseng* reduced the production of nitric oxide (NO) in mouse peritoneal macrophages. Ling *et al.*[8] demonstrated that the production of TNF-α in cultured human vascular endothelial cells could be reduced by *Panax notoginseng*, while Peng *et al.*[11] observed a significant decrease in the production of serum TNF-α in Sprague–Dawley rats following once-daily intraperitoneal injection of *Panax notoginseng*. In degenerated human discs, an increase in the levels of IL-1β and MMPs-1, 3, 7, and 13 were observed, with the magnitude of increase being associated with the severity of degeneration [12-14]. Additionally, Kohyama *et al.*[15] and Kang *et al.*[16] reported that human discs with herniation show increased production of NO, which might be associated with the induction of apoptosis in disc cells. TNF-α and IL-1β are implicated in the regulation of this catabolic process, while MMP-13 is a collagen-degrading enzyme [12]. The results of these studies suggest that *Panax notoginseng* might be useful for inhibiting the production of TNF-α, MMP-13, IL-1β and NO, which are associated with disc degeneration, and these effects could have caused the positive changes in disc height observed in this study. Further studies are required to elucidate the effects of *Panax notoginseng* on disc degeneration.

There are several limitations to this study. The rat tail model was adopted because of the tail’s special structure with an absence of vertebral posterior elements for convenient and accurate application of external loading, the model’s availability for biological analysis, and its biological and biomechanical properties, which are comparable to those of human lumbar discs [29-31]. However, it should be noted that there were notochordal cells in the mature rat caudal disc, which differ from the chondrocyte-like cells found in mature human discs [32,33]. Because the rat tail is not a weight-bearing spine, the biomechanical environment differs between rat caudal discs and human lumbar discs. Owing to differences in size and biomechanical environment between rat caudal and human discs, the deficit of nutrient supply in human discs might not have been observed in the rat caudal disc [33]. Moreover, the rat tail model used could only represent an acute model of disc compression. This might differ from the chronic condition of disc degeneration in humans [33]. Besides, the skin on rats’ tails is different from human skin, so the efficiency of intake of the herbal medicine might be different. Finally, stainless steel pins were inserted into the adjacent vertebral bodies of the target disc for the application of continuous static compression. These inserted pins might have affected the nutrient supply and the biomechanical environment of the disc.

## Conclusion

The *tienchi*–containing herbal plasters had no significant effect on the morphology of compressed intervertebral discs, but maintained disc heights in rats.

## Abbreviations

CM: Chinese Medicine; NP: Nucleus pulposus; AF: Annulus fibrosus; TNF-α: Tumor necrosis factor-α; IL-1β: Interleukin-1β; iNO: inducible nitric oxide;MMP-13: Matrix metalloproteinase-13; NO: Nitric oxide.

## Competing interests

The authors declare that they have no competing interests.

## Authors’ contributions

DHKC, AL, and MCPL designed the study. DHKC analyzed the data. AL conducted the experiments, and wrote the manuscript. FHT performed the radiography. All authors read and approved the final manuscript.
